# Perianal Abscess Following Excisional Hemorrhoidectomy in an Immunocompetent Patient

**DOI:** 10.7759/cureus.31292

**Published:** 2022-11-09

**Authors:** Manuel A Orellana Olmedo, Hanjoo Lee

**Affiliations:** 1 Surgery, Harbor University of California Los Angeles Medical Center, Torrance, USA; 2 Surgery, University of California Los Angeles David Geffen School of Medicine, Los Angeles, USA

**Keywords:** rectal discharge, perianal disease, colon & rectal surgery, immuno-competent, perirectal abscess

## Abstract

Hemorrhoidal disease is the third most common outpatient gastrointestinal diagnosis affecting more than four million patients annually. The management depends on the disease severity, and the treatment options range from lifestyle modification to excisional hemorrhoidectomy. Perianal abscess is an exceedingly rare complication following hemorrhoidectomy, with immunocompromised patients the most commonly affected. The rarity of this complication may be attributed to the natural immunologic process within the reticuloendothelial apparatus of the liver. The disease presentation of perianal abscess after hemorrhoidectomy and its management is unclear in the literature. We present the case of a 44-year-old immunocompetent male with grade II and III hemorrhoidal disease who underwent excisional hemorrhoidectomy that was complicated with perianal abscesses. The patient was successfully managed with incision and drainage with antibiotics. Surgeons should maintain a high index of suspicion for any sign of pelvic sepsis or a developing perianal abscess, particularly in immunocompromised patients.

## Introduction

In the United States, the hemorrhoidal disease is the third most common outpatient gastrointestinal diagnosis affecting more than four million patients annually [1]. This pathology is often associated with perianal edema, pain, prolapse of anal mucosa, discharge, bleeding, and irritation of the perianal skin [2].

Various treatment options are available, ranging from non-operative strategies to surgical excision. Lifestyle modification with adequate daily hydration and fiber intake is used as the first-line therapy; moreover, excisional hemorrhoidectomy is the gold standard for grades III and IV and in cases where the office-based procedure is unfeasible [3].

Complications for excisional hemorrhoidectomy are well described. Urinary retention and anal stenosis are the most common early and late complications, respectively. Anal fistula and perianal abscess are reported in less than 0.5% of the cases [4]. Excisional hemorrhoidectomy involves creating a surgical wound close to the rectum, an area of the human body with the highest bacterial burden [5]. Despite this, submucosal abscess and sepsis development are unusual, with only a handful of case reports and series [6].

The rarity of a perirectal abscess following hemorrhoidectomy inevitably means its management is not well described. Herein we describe a case of a 44-year-old male patient who presented with two perirectal abscesses following excisional hemorrhoidectomy treated with incision and drainage.

## Case presentation

A 44-year-old male with no other medical history and circumferential grade II and III bleeding hemorrhoidal disease underwent excisional hemorrhoidectomy at the right posterior, right anterior, and left posterior regions. The complete excision of hemorrhoidal diseases could put the patient at risk for anal stricture, therefore the small residual disease was treated with hemorrhoidal banding. He presented to the emergency department (ED) on a post-operative day (POD) 3 with subjective fever and chill. He was tachycardic on arrival, which resolved with fluid resuscitation. He remained afebrile. A digital rectal exam was notable for a well-healing hemorrhoidectomy site without any sign of infection. Urinalysis was unremarkable, and the white blood cell count was within the normal range. The patient was discharged home from the ED. On POD 7, a phone interview was done for a symptom check. The patient reported good improvement in pain without fever and chills and normal passing of soft bowel movements daily.

**Figure 1:**
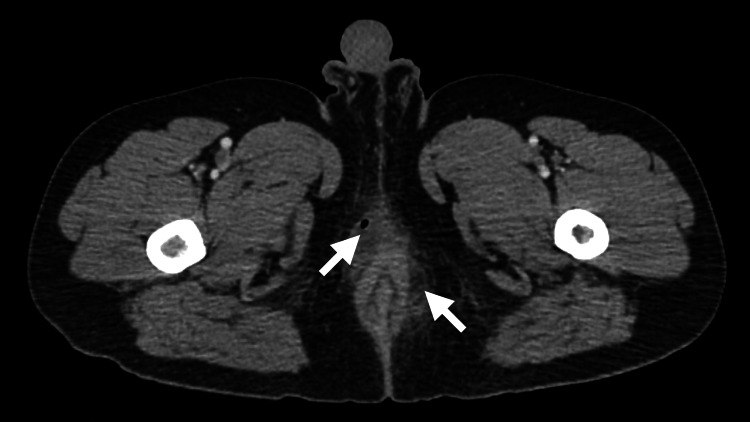
CT image of the perianal abscesses in the right anterior and left lateral regions.

On POD 12, he returned to the ED with severe anal pain and purulent drainage from the anus. On physical examination, copious purulent drainage from the anal canal was found in the left lateral hemorrhoidectomy site. A CT image of the pelvis showed perianal abscesses in the right anterior and the left lateral region (Figure 1). See Table 1 below for laboratory values.

**Table 1:** Laboratory values. The patient came in on the first ED visit on POD 3 and was discharged home after confirming no signs of pelvic sepsis. The patient came back on POD 12 with signs of pelvic sepsis associated with a pelvic abscess and then was emergently transferred to the operating room for incision and drainage. WBC, white blood cell; RBC, red blood cell; HGB, hemoglobin; HCT, hematocrit; MCV, mean corpuscular volume; MCH, mean corpuscular hemoglobin; MCHC, mean corpuscular hemoglobin concentration; RDW, red cell distribution width; MPV, mean platelet volume; POD, postoperative day; ED, emergency department

	Pre-op	First ED visit (POD 3)	Second ED visit (POD 12)
WBC	6.6 K/cumm	9.3 K/cumm	13.6 K/cumm
RBC	4.82 M/cumm	4.72 M/cumm	4.64 M/cumm
HGB	7.7 g/dL	7.8 g/dL	7.5 g/dL
HCT	27.80%	27.10%	26.80%
MCV	57.7 fL	57.5 fL	57.8 fL
MCH	15.9 pg	16.5 pg	16.1 pg
MCHC	27.5 g/dL	28.7 g/dL	27.9 g/dL
RDW	22.50%	22.20%	22.50%
Platelet	369 K/cumm	379 K/cumm	664 K/cumm
MPV	8.3 fL	8.4 fL	8.3 fL
Neutrophils		77.90%	84.00%
Lymphocytes		10.60%	8.70%
Eosinophils		7.50%	5.70%
Basophils		3.80%	1.10%
Neutrophils absolute		0.20%	0.50%
Lymphocytes absolute		7.2 K/cumm	11.4 K/cumm
Monocytes absolute		1.0 K/cumm	1.2 K/cumm
Eosinophils absolute		0.4 K/cumm	0.8 K/cumm
Basophils absolute		0.0 K/cumm	0.1 K/cumm

White blood cell count was elevated to > 13,000/mm^3^. Testing for HIV was negative. An intravenous antibiotic was initiated, and he underwent an urgent examination under anesthesia, incision, and drainage of the perianal abscesses. 10cc of purulent material was drained from the left lateral abscess cavity, and minimal drainage was noted from the right anterior region. He was discharged home on POD 1 with a 14-day course of oral antibiotics. A one-week postop follow-up clinic visit showed well-healing incision and drainage sites with complete resolution of symptoms.

## Discussion

Post-operative pelvic sepsis following hemorrhoidectomy is a rare complication reported only by scant case reports; this is remarkable given the virulent bacteria that reside in these areas [7]. One possible explanation for this phenomenon is the natural immunologic process within the portal system, as the reticuloendothelial apparatus clears bacteria from the liver [8]. Symptoms of pelvic sepsis after hemorrhoidectomy include perineal pain, abdominal tenderness, leukocytosis, fever, rectal discharge, necrosis, and even septic shock. Most of these affected patients were successfully managed with antibiotics, drainage, and necrosectomy; one case needed intensive care unit admission, and one resulted in mortality [6, 9].

The pathophysiology of post-operative perianal abscess after excisional hemorrhoidectomy is not well understood due to the rarity of this event. However, it is presumed to be caused by the breakdown of normal anal mucosa and translocation of the natural enteric bacterial flora including *Escherichia coli*, *Klebsiella pneumoniae*, *Enterococcus* and *Bacteroides fragilis* resulting in transient bacteremia. Transient bacteremia often presents with fever, diaphoresis, chills, and rigor [10-11]. These clinical manifestations may precede the development of perianal abscess, as seen in this case scenario. A high index of suspicion for overwhelming pelvic sepsis and readiness to return to the operating room for an urgent exam under anesthesia is prudent. If symptomatic bacteremia is transient, as in this case scenario, the authors recommend initiating oral broad-spectrum antibiotics with close outpatient follow-up.

Immunocompromised patients are the most commonly affected group that may develop bacteremia and perianal abscess following hemorrhoidectomy [12]. Therefore, it is prudent to evaluate the use of immunosuppressants and immunocompromised states such as human immunodeficiency virus (HIV) positivity or agranulocytosis [13].

Finally, several technical aspects must be considered; in this case, the patient was managed by traditional Ferguson's closed hemorrhoidectomy [14]. A ligating suture was placed in the apex of the hemorrhoidal pile. While adequate ligation of the hemorrhoidal pedicle is often necessary, authors caution against full-thickness sutures in this location to avoid "seeding" the perianal tissue with virulent bacteria of the anal canal. In addition, avoiding excessive use of suture materials and tissue removal would be prudent to minimize trauma to the tissue.

Management of abscesses after hemorrhoidectomy should follow the standard incision and drainage technique of perianal abscesses. In this case, the incisions were made at the sites of most fluctuance, and the abscess cavities were allowed to drain. Although there is no substantial evidence to support continued antibiotic use after drainage of perianal abscesses [15], authors recommend continued antibiotics in the immediate post-operative period in these rare scenarios, especially if systematic signs of infection such as fever are present.

## Conclusions

Perianal abscess following excisional hemorrhoidectomy is a complication out of the ordinary that requires a return to the operating room for an examination under anesthesia and drainage. Given that most of the reported cases of perianal abscess after hemorrhoidectomy involved immunocompromised patients, it is imperative to test patients for the use of immunosuppressants or HIV. Therefore, when performing hemorrhoidectomy in immunocompromised patients, the surgeon should maintain a high index of suspicion for any sign of impending pelvic sepsis or a developing perianal abscess. Finally, treatment with broad-spectrum antibiotics and prompt infectious source control with incision and drainage is warranted.
